# Estrogens Modulate Somatostatin Receptors Expression and Synergize With the Somatostatin Analog Pasireotide in Prostate Cells

**DOI:** 10.3389/fphar.2019.00028

**Published:** 2019-02-15

**Authors:** Valentina Rossi, Erika Di Zazzo, Giovanni Galasso, Caterina De Rosa, Ciro Abbondanza, Antonio A. Sinisi, Lucia Altucci, Antimo Migliaccio, Gabriella Castoria

**Affiliations:** ^1^Dipartimento di Medicina di Precisione, Università degli Studi della Campania “Luigi Vanvitelli”, Naples, Italy; ^2^Dipartimento di Scienze Mediche, Chirurgiche, Neurologiche, Metaboliche e dell’Invecchiamento, Università degli Studi della Campania “Luigi Vanvitelli”, Naples, Italy

**Keywords:** prostate cancer, estrogens, somatostatin analogs, somatostatin receptors, apoptosis, EMT, migration

## Abstract

Prostate cancer (PC) is one of the most frequently diagnosed cancers and a leading cause of cancer-related deaths in Western society. Current PC therapies prevalently target the functions of androgen receptor (AR) and may only be effective within short time periods, beyond which the majority of PC patients progress to castration-resistant PC (CRPC) and metastatic disease. The role of estradiol/estradiol receptor (ER) axis in prostate transformation and PC progression is well established. Further, considerable efforts have been made to investigate the mechanism by which somatostatin (SST) and somatostatin receptors (SSTRs) influence PC growth and progression. A number of therapeutic strategies, such as the combination of SST analogs with other drugs, show, indeed, strong promise. However, the effect of the combined treatment of SST analogs and estradiol on proliferation, epithelial mesenchyme transition (EMT) and migration of normal- and cancer-derived prostate cells has not been investigated so far. We now report that estradiol plays anti-proliferative and pro-apoptotic effect in non-transformed EPN prostate cells, which express both ERα and ERβ. A weak apoptotic effect is observed in transformed CPEC cells that only express low levels of ERβ. Estradiol increases, mainly through ERα activation, the expression of SSTRs in EPN, but not CPEC cells. As such, the hormone enhances the anti-proliferative effect of the SST analog, pasireotide in EPN, but not CPEC cells. Estradiol does not induce EMT and the motility of EPN cells, while it promotes EMT and migration of CPEC cells. Addition of pasireotide does not significantly modify these responses. Altogether, our results suggest that pasireotide may be used, alone or in combination with other drugs, to limit the growth of prostate proliferative diseases, provided that both ER isoforms (α and β) are present. Further investigations are needed to better define the cross talk between estrogens and SSTRs as well as its role in PC.

## Introduction

Prostate cancer represents the most common type of cancer among males in Western society and it is commonly considered a “hormone-dependent cancer”. Sex steroids, mainly the androgens, control, indeed, its initiation and progression. PC is initially an androgen-dependent disease and the ADT still represents the major pharmacological option at this tumor stage (*reviewed* in Ryan and Tindall, 2011). ADT, however, frequently fails, and the disease progresses to an androgen-independent state, also known as CRPC. At this stage, current therapies scantly improve patient’s survival. New pharmacological approaches are, therefore, needed to limit or inhibit PC growth and spreading (*reviewed* in Castoria et al., 2017).

Estrogens are involved in PC etiology and progression. Epidemiologic and clinical evidence links the sustained exposure to estrogens with increased risk of developing PC. Nevertheless, the mechanism by which estrogens induce prostate cancerogenesis and foster PC progression has not been fully identified (*reviewed* in Di Zazzo et al., 2016). As it occurs in BC (Huang et al., 2007) and benign prostatic hyperplasia (Shao et al., 2014), estrogens might control EMT, thereby leading to PC invasiveness and metastasis.

ERs, α or β, mediate the estrogen effects in target cells and normal human prostate expresses both ER isoforms. It is generally accepted that ERα mediates the adverse effects (i.e., proliferation and inflammation) induced by estrogens, while ERβ mediates the protective and anti-apoptotic estrogen effects in PC. However, the concept that ER α and β mutually antagonize their action in PC is debated, since cellular responses might depend on the cross talk between the two receptors occurring at transcriptional (Madak-Erdogan et al., 2013; Karamouzis et al., 2016) or non-transcriptional (Rossi et al., 2009) level. Furthermore, the ratio between the two ER isoforms, the fluctuations in ligand concentration, the presence of endogenous inhibitors and the availability of transcriptional co-regulators might differently modulate the ERα- or β-mediated responses in target cells (Warner et al., 2017). Conflicting findings on the role of ERα or β in PC continue to emerge (Di Zazzo et al., 2018). High ERβ protein levels are associated, for instance, with EMT in PC cells and a worse prognosis in PC patients (Nanni et al., 2009). In contrast, specific activation of ERβ seems to maintain an epithelial phenotype and represse PC cell invasiveness (Mak et al., 2010). It seems clear that additional studies are needed to disclose these discrepancies as well as the exact role of ERα or β in EMT and PC progression (*reviewed in* Montanari et al., 2017).

The regulatory neuropeptide, SST induces the growth arrest and apoptosis in neuroendocrine and inflammatory cells (Patel, 1999). By acting on both pituitary hormone release and prostate gland, SST exerts a pro-apoptotic effect in prostate cells. Moreover, SST analogs exhibit a potent anticancer activity in cultured cells as well as *in vivo* models. As such, therapeutic strategies, based on the combination of SST analogs with other antineoplastic drugs, appear very promising.

Somatostatin action is mediated by five specific high-affinity G-protein coupled receptors SSTR1-5, which belong to the seven-*trans*-membrane segment receptor superfamily and are expressed in a wide variety of solid tumors, including PC (Møller et al., 2003; Msaouel et al., 2009). All of the five SSTRs can be detected in the prostate epithelial cells or PC tissues. SSTR-2 is expressed in normal prostate tissue and in a subset of highly invasive PC, while SSTR-1 and SSTR-5 are prevalently expressed in PC (Sinisi et al., 1997; Halmos et al., 2000; Lattanzio et al., 2013). Therefore, SSTRs represent a target for PC therapies, although the mechanism of their action as well as their cross talk with steroid receptors is still unclear. Estrogens up-regulate SSTR expression in BC cells as well as in goldfish pituitary and cerebral tissues (Djordjijevic et al., 1998; Kimura et al., 2001; Canosa et al., 2003). However, the effect of the combined treatment of SST analogs and estradiol on proliferation, EMT and migration of normal and PC cells has not been investigated so far.

In the present study we have used two *in vitro* cultured cell models, the prostate epithelial EPN cell line, which expresses AR and both the ER isoforms (α and β) and a PC-derived cell line, CPEC, which expresses low levels of both ERβ and AR, but lacks ERα (Sinisi et al., 2002; Rossi et al., 2009 and present paper). In these cell lines, we have compared the effects of estradiol and pasireotide, alone or in combination, on SSTR1, SSTR2, SSTR3 and SSTR5 expression at both mRNA and protein levels. At last, we have assessed the effect of these treatments on cell viability, EMT and migration by analyzing cell cycle, apoptosis as well as EMT markers and cell motility in the aforementioned models.

## Materials and Methods

### Cell Culture and Chemicals

Cell media and supplements were supplied by Invitrogen. EPN and CPEC cell lines were derived from epithelial normal prostate tissue or PC, respectively. Isolation and characterization of both cell lines has been previously described (Sinisi et al., 2002; Pasquali et al., 2006; Rossi et al., 2009). Cells were maintained at 37°C in humidified 5% CO_2_ atmosphere and cultured in Nutrient Ham’s F12, supplemented with 3% fetal calf serum (FCS) and antibiotics. Cells (at 70% confluence) were cultured for 5 days in phenol red-free Minimum Essential Medium, containing antibiotics and 1% charcoal-treated FCS, to remove steroids and minimize serum effect. The culture medium was changed every day. Cells were left untreated or treated for the indicated times with estradiol (Sigma-Aldrich; at 20 nM final concentration), in the absence or presence of pasireotide (Novartis; at 0,1 μM final concentration), before harvesting. Parallel cells were treated with vehicle or pasireotide alone, as a control. When indicated, the cells were treated with the ERβ agonist, DPN (Tocris; at 3 nM final concentration) or the ERα agonist, PPT (Tocris; at 3 nM final concentration). Human BC-derived cells MCF-7 cells and PC-derived LNCaP cells were from Cell Bank Interlab Cell Line Collection (ICLC, Genova, Italy). Cells were maintained at 37°C in humidified 5% CO_2_ atmosphere. MCF-7 cells were cultured in DMEM supplemented with glutamine (2 mM), penicillin (100 U/ml), streptomycin (100 U/ml), insulin (6 ng/ml), hydrocortisone (3.75 ng/ml) and 5% FBS. LNCaP cells were cultured in RPMI-1640 supplemented with 10% FBS, glutamine (2 mM), penicillin (100 U/ml), streptomycin (100 U/ml), sodium pyruvate (1 mM) and non-essential amino acids (10 mM). Media and supplements were from Gibco. The cell lines employed throughout the paper were routinely monitored for Mycoplasma contamination, expression of steroid receptors and steroid responsiveness, as reported (Castoria et al., 2014).

### RNA Isolation and Semi-Quantitative Reverse-Transcription PCR (RT-PCR) Analysis

At the indicated times, total RNA (1 μg) was extracted from EPN and CPEC cells and then purified, using TRIzma Reagent (Sigma-Aldrich) as reported (Porcile et al., 2014). RNA samples were eluted in 50 μl of water treated with diethyl-pyrocarbonate and stored at -80°C. The quality of RNA was assessed by gel electrophoresis in denaturing conditions and evaluation of 260/280 nm and 260/230 nm absorbance ratios. RNA samples with absorbance ratio 260/280 nm lower than 1.9 or with absorbance ratio 260/230 lower than 2.2 were discarded. RNA samples were then treated with 40U of RNAse-free DNAse-I (Boehringer Mannheim) for 45 min at 37°C. To exclude the presence of genomic DNA, PCR amplification was performed on RNA samples not reverse-transcribed, too. MMLV-Reverse Transcriptase and random primers (Bio-Rad Laboratories) were used to reverse-transcribe total RNA. cDNAs amplification was performed by RT-PCR with specific primers set for Bcl-2, GAPDH (Pasquali et al., 2006; Rossi et al., 2009, 2011), c-Myc (Gazzerro et al., 2006), SSTR1, SSTR2, SSTR3 and SSTR5 (Pasquali et al., 2008) transcripts, using JF buffer (30 mM Tris base, 8 mM HEPES base, 20 mM K glutamate, 60 mM NH4 acetate, 2 mM DTT, 8% glycerol, 1.5 mM MgCl2, 0.2 mM dNTPs). RT-PCR analysis was done as previously described (Abbondanza et al., 2012). Calibration curve for RT-PCR analysis was obtained by serial dilutions of cDNA, which were analyzed to verify the linearity of the PCR reaction. Data obtained by RT-PCR fell within the linearity range. cDNAs were amplified in triplicate and reaction was done using a thermal cycler (Eppendorf). Amplified products were analyzed by electrophoresis, using 2% agarose gel. Gel images were acquired by the Gel DOC XR System platform (Bio-Rad Laboratories).

### Cell Cycle Analysis

Growing EPN and CPEC cells at 70% confluence were made quiescent and then left unstimulated or stimulated for the indicated times with estradiol (20 nM), in the absence or presence of pasireotide (0,1 μM). When indicated, pasireotide (0,1 μM) was used alone, as control. Cells were harvested in PBS containing 5 mM EDTA. Cell pellets were washed twice with PBS and then centrifuged at 1,200 rpm. Permeabilization was performed by incubating 10^6^ cells in 0,5 ml of flow cytometry analysis (FACS) permeabilizing solution, which contains RNAses and propidium iodide (Beckton Dickinson). Incubation was done for 2 h at 4°C in the dark. Flow cytometry data were collected using a FACScalibur instrument and analyzed by CELL QUEST software (Beckton Dickinson).

### Epithelial Mesenchyme Transition (EMT) Analysis, BrdU Incorporation, Scratch Wound Migration Analysis and Contrast-Phase Microscopy

Growing EPN or CPEC cells were plated (4 × 10^4^ cells) in culture multiwell plates (6 plates). After 24 h, the cells were made quiescent and then left unstimulated or stimulated for 48 h with 20 nM estradiol, in the absence or presence of the indicated compounds. Pasireotide was used at 0,1 μM. Cell lysates were then prepared, as below described. Parallel multiwell plates were used for wound scratch analysis, which was done as reported (Castoria et al., 2011). Briefly, cells at 90% confluence were made quiescent and then scratch wounded with sterile pipette tip. They were left untreated or treated for 48 h with estradiol (20 nM), in the absence or presence of pasireotide (0,1 μM). Parallel cells were left untreated or treated with pasireotide alone (0,1 μM) or the ERβ agonist DPN (3 nM) or the ERα agonist PPT (3 nM). Cytosine arabinoside (Sigma; at 20 μM final concentration) was included in the cell medium to avoid cell proliferation. The proliferation rate of cells was monitored by BrdU incorporation analysis in immunofluorescence microscopy (Pagano et al., 2004), using a DMBL Leica (Leica) fluorescent microscope equipped with HCX PL Apo 63x oil objective. Morphological changes and wound scratch analysis were analyzed by contrast-phase microscopy using a DMIRB (Leica) microscope, equipped with X-Plan 10x or HCX PL Fluotar 40× or 63× objectives. Images were acquired using a Leica DFC 450C digital camera and processed using Leica Suite software, as reported (Di Donato et al., 2015a, 2018). Images are representative of 3 different experiments. When indicated, the wound gap was calculated using Image J sofware and expressed as % of the decrease in the wound area.

### Lysates, Antibodies and Western Blot

Epithelial mesenchyme transition markers were analyzed by Western blot technique. Briefly, unstimulated or stimulated cells were harvested in PBS containing 5 mM EDTA. Cell pellets were washed twice by centrifugation with PBS at 1,200 rpm and lysate proteins were prepared as reported (Castoria et al., 2011). SDS-PAGE and Western blot analysis were done according to the same report. Mouse monoclonal anti-cytokeratin (C11; Santa Cruz) and rabbit polyclonal anti-vimentin (H-84; Santa Cruz) antibodies were used to detect cytokeratin and vimentin, respectively. The mouse monoclonal anti E-cadherin (clone HECD-1; Abcam) antibody was used to detect E-cadherin. Unless otherwise stated, lysate preparation, SDS-PAGE and Western blot analysis (WB) were done as described elsewhere (Donini et al., 2012). A new panel of monoclonal antibodies to human SSTRs (MoAb Y/SSTR1, MoAb Y/SSTR2, MoAb Y/SSTR3, MoAb Y/SSTR5) was used for the primary immunoreaction. Antibodies were raised against a peptide sequence of SST-binding domains endowed within the extracellular loop of the SSTRs. Antibody specificity was assessed by Western blot analysis of proteins extracted from normal pancreas tissue (Supplementary Figure S1). ERα and ERβ were detected as described (Castoria et al., 2014), using the rabbit polyclonal anti-ERalpha antibody (543; Santa Cruz) or the rabbit polyclonal anti-ERβ antibody (UBI). c-Myc was revealed as reported (Castoria et al., 2004), using the anti-c-Myc mouse monoclonal antibody (clone 9E10; Zymed Laboratories). Bcl-2 was detected as reported (Perillo et al., 2000), using the mouse monoclonal anti Bcl-2 antibody (clone Bcl-2-100; TermoFisher). Caspase-3 activation was detected using the rabbit polyclonal anti-cleaved caspase-3 antibody (Millipore). Tubulin was detected using mouse monoclonal anti-tubulin antibody (Sigma-Aldrich). Immunoreactive proteins were revealed using the ECL system (GE Healthcare). When indicated, densitometry analysis was done by Image J software (ImageJ, U. S. National Institutes of Health, Bethesda, MD, United States^1^), using the “Gel Plot” plug-in.

### Statistical Analysis

Statistical analysis was performed by using ANOVA for multiple comparisons and paired *t*-test to compare individual cell responses to treatment. *P* < 0.05 values were considered significant.

## Results

### Estradiol Effect on SSTRs in EPN and CPEC Cells

**FIGURE 1:**
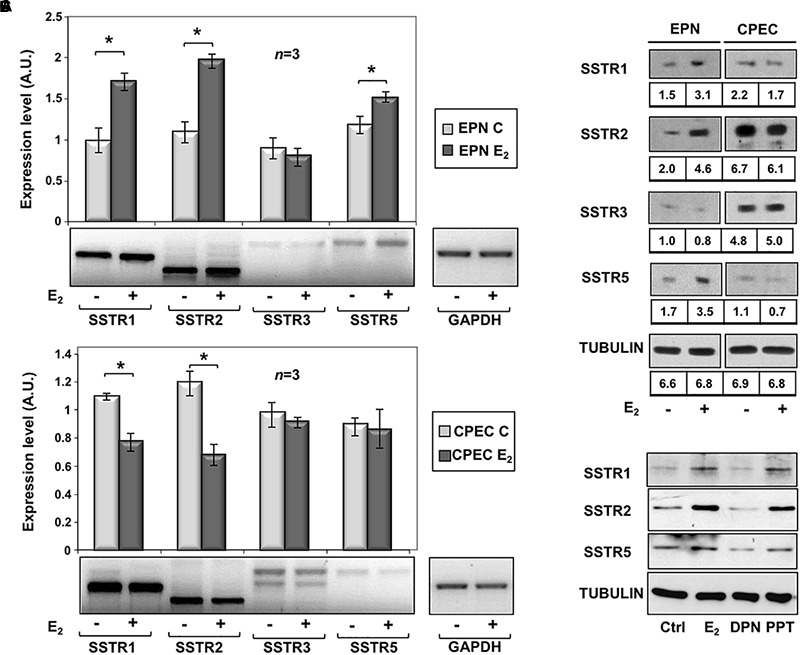
Effect of estradiol on SSTRs mRNA and protein expression levels. RT-PCR analysis of SSTR1, 2, 3 and 5 mRNA levels in EPN **(A)** or CPEC **(B)** cells untreated or treated for 24h with 20 nM estradiol (E_2_) was done. In panel **(A,B)**, densitometry analysis of agarose gel electrophoresis is shown. The expression level is indicated as fold changes over the basal conditions (A.U.). Histograms represent the averages (+/– standard error) from 3 independent experiments, normalized for the expression of the control housekeeping gene GAPDH (^∗^indicates *p* < 0.05 for each gene *versus* its untreated control). Means and SEMs are shown. *n* represents the number of experiments. ^∗^*p* < 0,05. Insets are representative from one experiment in **A** or **B**. In panel (**C**, upper section), the Western blot analysis of lysate proteins from EPN and CPEC cell lines untreated or treated with estradiol (20 nM; E_2_) for 48 h was done. Lysate proteins (1 mg/ml) were resolved by SDS–PAGE, transferred to PVDF membrane and probed with anti-SSTRs or anti-tubulin antibodies. Inset in panel **(C)**, densitometry analysis of SSTRs was done in two different experiments using the NIH/Image J software. Results were expressed as relative change over the basal level of each SSTR isoform. In panel (**C**, lower section), the Western blot analysis of lysate proteins from EPN cells untreated or treated with estradiol (20 nM; E_2_) or PPT (3 nM) or DPN (3 nM) for 48 h was done. Lysate proteins (2 mg/ml) were resolved by SDS–PAGE, transferred to nitrocellulose membrane and probed with the antibodies against the indicated proteins. In panel (**C**, upper and lower sections), the Western blot analysis of tubulin is shown, as loading control.

To assess the effects of estradiol on SSTRs expression, we used EPN, which express ERα and ERβ and CPEC, which only express low levels of ERβ (Supplementary Figure S2 and Rossi et al., 2011). The cells were left untreated or treated with estradiol (20 nM) for 24 h. Figure 1A and inset show that estradiol treatment of EPN cells resulted in a significant increase in SSTR1, 2 and 5 mRNA, as compared to untreated cells. Hormone treatment did not affect SSTR3 gene expression. In contrast, estradiol treatment of CPEC cells significantly reduced SSTR1 and 2 mRNAs. A negligible hormonal effect was observed on SSTR3 and SSTR5 gene expression (Figure 1B and inset). The Western blot analysis and densitometry quantification of bands show that estradiol treatment increased the expression of SSTR1, 2 and 5 in EPN cells (Figure 1C and inset). A weak, but significant reduction of SSTR1 and SSTR2 protein levels was detectable in CPEC cells, in the absence of significant changes in SSTR3 protein levels (Figure 1C and inset). The observation that estradiol down-regulates SSTR5 protein levels (Figure 1C and inset) in the absence of significant changes in gene expression might be due to post-translational modification and degradation of SSTR5 induced by the hormone. SSTRs undergo, indeed, phosphorylation at different sites with the subsequent degradation (Csaba et al., 2012) and estradiol activates multiple kinases in target cells (Migliaccio et al., 2007a,b; Castoria et al., 2008).

To further evaluate the role of hormone regulation in SSTR expression, we challenged quiescent EPN cells with estradiol, or PPT and DPN ligands, which specifically bind ERα or ERβ, respectively. The Western blot analysis in Figure 1C (lower section) shows that the increase in expression of SSTR1, 2 and 5 is mainly due to ERα activation by PPT ligand in EPN cells. The down regulation of these receptors in CPEC cells, which only express ERβ, is very likely caused by ERβ activation. We did not use siRNA approach, since a cross talk between ERα and ERβ regulates the expression of each other in PC cells (Pisolato et al., 2016).

In sum, experiments in the Figure 1 shows that estradiol up-regulation of SSTR1, SSTR2 and SSTR5 occurs mainly through ERα activation in EPN cells. The absence ERα is likely responsible for estradiol-induced down-regulation of SSTR1, SSTR2 and SSTR5 in CPEC cells.

### Effect of Estradiol and Pasireotide in EPN and CPEC Cells: Studies on Cell Cycle Progression and Caspase-3 Activation

**FIGURE 2:**
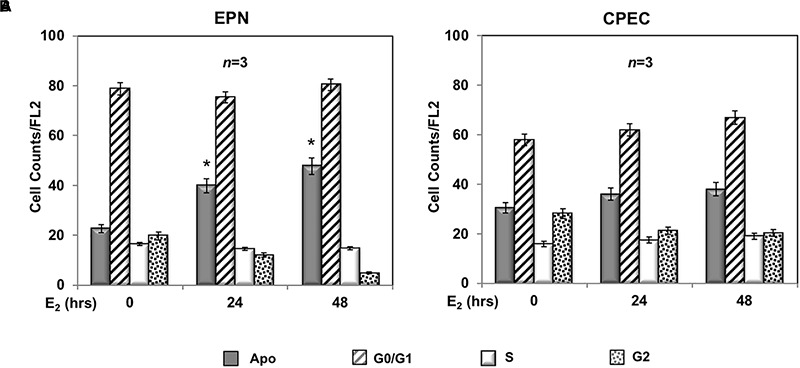
Effect of estradiol on cell cycle. Flow cytometry analysis of propidium iodide-labeled EPN **(A)** or CPEC **(B)** cells was done, as described in Methods. Quiescent cells were left under basal conditions or treated with estradiol (20 nM; E_2_) for the indicated times. Histograms represent the % counts/FL2 areas of labeled cells in the different cell cycle phases after 24 or 48 h of hormonal treatment. Means and SEMs are shown. *n* represents the number of experiments. ^∗^*p* < 0,05 for each experimental point vs. the corresponding untreated control.

A 24- and 48-h response of propidium-iodide incorporation analysis was then performed to test the role of estradiol in EPN and CPEC cell proliferation. The FACS analysis in Figure 2A shows that 24 and 48 h treatment of EPN cells with estradiol induced in both conditions a significant increase of pre-G1 apoptotic cells associated with a persistently elevated number of cells in G0/G1 phases and a low amount of cells in S-phase. In contrast, estradiol treatment of CPEC cells did not significantly affect the number of apoptotic cells, with cells persistently arrested in G0/G1 phase (Figure 2B).

**FIGURE 3:**
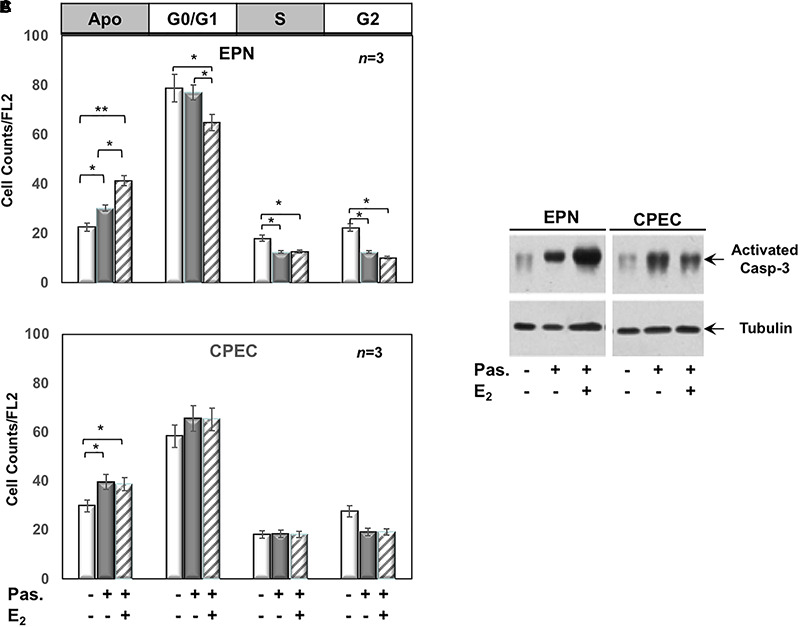
Effect of both estradiol and pasireotide on cell cycle. Flow cytometry analysis of propidium iodide-labelled EPN **(A)** or CPEC **(B)** was done, as described in Methods. In panel **(A,B)**, quiescent cells were left under untreated or treated for 24 h with 0,1 μM pasireotide, in the absence or presence of 20 nM estradiol. Histograms in panel **(A,B)** represent the % counts/FL2 areas of labeled cells in the different cell cycle phases after the indicated treatments. Means and SEMs are shown. *n* represents the number of experiments. ^∗^*p* < 0,05; ^∗∗^*p* < 0,01. In panel **(C)**, quiescent EPN (left section) or CPEC (right section) cells were left untreated or treated for 24 h with 0,1 μM pasireotide, in the absence or presence of 20 nM estradiol. Lysate proteins were separated by SDS-PAGE, transferred to PDVF membrane and filters were then analyzed by Western blot, using the anti-activated caspase 3 or anti-tubulin antibodies.

Twenty-four h treatment of EPN (Figure 3A) and CPEC (Figure 3B) cells with 0,1 μM of the SSTR-specific agonist, pasireotide, significantly increased the number of apoptotic cells. Co-treatment with estradiol reinforced the pro-apoptotic effect of pasireotide in EPN cells, with a reduction in the number of cells in S-phase (Figure 3A). In contrast, co-treatment of CPEC cells with estradiol and pasireotide (0,1 μM) did not enhance the number of apoptotic cells (Figure 3B). By prolonging the time of treatment (48 h) and by increasing the concentration of pasireotide (1μM), we did not observe any further increase in the number of apoptotic cells (not shown). The strong stability of pasireotide, together with its high affinity for all SSTRs (*reviewed in* Feelders et al., 2013) might be responsible for its efficacy in inducing the apoptotic response at very low concentration (0,1 μM) and within a relatively short time frame (24h). Lastly, the Western blot analysis of activated caspase-3 in Figure 3C confirmed the data obtained by FACS analysis of EPN and CPEC cells.

In sum, Figure 2, 3 show that EPN cells challenged with estradiol undergo a robust apoptotic response, while CPEC cells remain arrested in G0/G1. Pasireotide induces apoptotic death in both EPN and CPEC cells, and the simultaneous treatment with estradiol enhances the pasireotide effect only in EPN cells.

### Effect of Estradiol and Pasireotide in EPN and CPEC Cells: Regulation of Bcl-2 and Myc Gene Expression and Protein Levels

**FIGURE 4:**
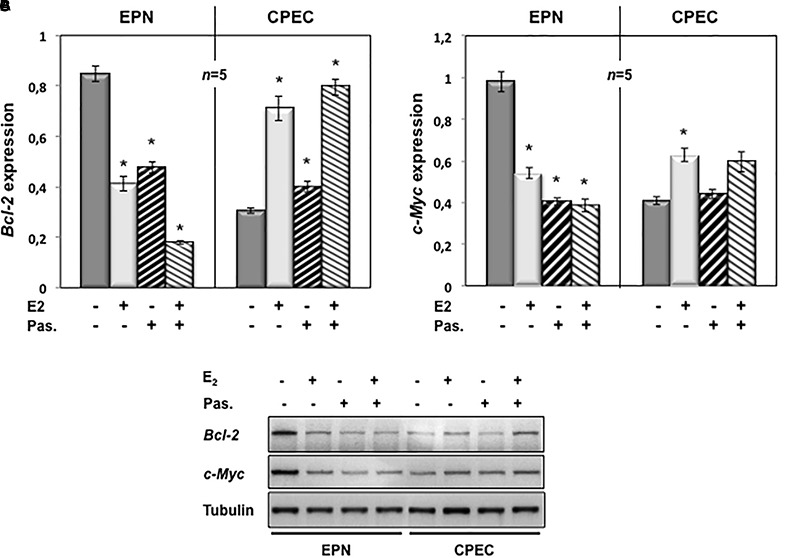
Effect of both estradiol and pasireotide on Bcl-2 and Myc mRNA expression levels. EPN or CPEC cells were untreated or treated for 24 h with estradiol (20 nM; E_2_), in the absence or presence of 1 μM pasireotide. RT-PCR analysis of Bcl-2 **(A)** and c-Myc **(B)** mRNA levels was done. Densitometry analysis of agarose gel electrophoresis was done. The expression level is indicated as fold changes from basal conditions (A.U.). Histograms represent the averages (+/- standard error) from five independent experiments, normalized for the expression of the control housekeeping gene GAPDH. Means and SEMs are shown. ***n*** represents the number of experiments. ^∗^*p* < 0.05 for each experimental point *versus* the corresponding untreated control. In panel **(C)**, EPN or CPEC cells were untreated or treated for 24 h with estradiol (20 nM; E_2_), in the absence or presence of 1 μM pasireotide. Lysate proteins were prepared, electrophoretically separated by SDS-PAGE and transferred to PDVF membrane. Filters were immune-blotted using anti-Bcl-2 or anti-c Myc or anti-tubulin antibodies.

To evaluate the putative intracellular targets responsible for the observed effects of estradiol and pasireotide on cell cycle, we analyzed by semi-quantitative RT-PCR the Bcl-2 gene expression in both EPN and CPEC cells treated with estradiol and pasireotide, alone or in combination. Estradiol induced a down-regulation of Bcl-2 gene expression in EPN cells and pasireotide co-treatment enhanced this effect (left panel in Figure 4A). In contrast, estradiol induced an increase in Bcl-2 mRNA, and simultaneous treatment with pasireotide enhanced such effect in CPEC cells (right panel in Figure 4A).

We next analyzed c-Myc gene expression levels. Twenty-nM estradiol induced a down-regulation of c-Myc mRNA in EPN cells and co-treatment with pasireotide reinforced the estradiol effect (left panel in Figure 4B). Estradiol, alone or in combination with pasireotide, induced a weak, but significant increase of c-Myc mRNA in CPEC cells (right panel in Figure 4B). Similar findings were observed by Western blot analysis for Bcl-2 and c-Myc protein levels (Figure 4C), although the synergic effect of estradiol and pasireotide was more evident by gene expression analysis. The slight difference we observe might due to modification of Bcl-2 and c-Myc occurring at post-translational level.

These findings indicate that estradiol and pasireotide have a synergic effect in decreasing Bcl-2 and c-Myc gene expression in EPN cells, while they increase such responses in CPEC cells.

### Effect of Estradiol and Pasireotide on EMT and Migration of EPN and CPEC Cells

**FIGURE 5:**
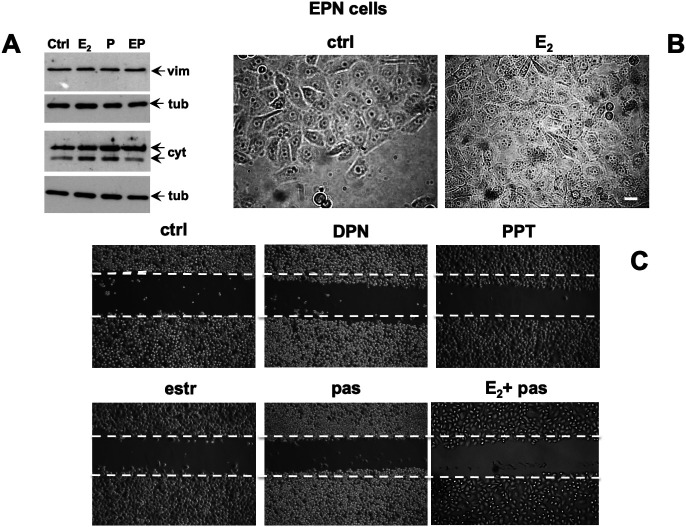
Effect of estradiol and pasireotide on EMT, morphology and motility of EPN cells. Quiescent EPN cells were untreated or treated for 48 h with the indicated compounds. Estradiol was used at 20 nM, pasireotide at 0.1 mM, PPT and DPN both at 3 nM. In panel **(A)**, lysate proteins (2 mg/ml) were prepared, separated by SDS-PAGE and transferred to nitrocellulose membrane. Filters were immune-blotted using the antibodies against the indicated proteins. The blots are representative of two different experiments. In panel **(B)**, the cells were analyzed for morphological changes using contrast-phase microscopy. Bar, 10 mM. In panel **(C)**, the cells were wounded and then left unstimulated or stimulated with the indicated compounds. Cytosine arabinoside (20 mM) was added to the cell medium to avoid cell proliferation. Contrast-phase images in panel **(B,C)** are representative of 3 different experiments.

Conflicting findings on the role of ERα or β in EMT of prostate cells and PC progression have been reported (Montanari et al., 2017). We then investigated by Western blot analysis the effect of estradiol, or pasireotide or combination of both in EMT of EPN cells (Figure 5A). Quiescent cells were left unchallenged or challenged for 48h with the indicated stimuli and lysate proteins were analyzed for expression of vimentin, E-cadherin and cytokeratins. Regardless of stimuli, the Western blots in Figure 5A shows that no significant changes in vimentin and E-cadherin levels were observed in EPN cells. Estradiol or pasireotide slightly increased the cytokeratin levels. Noticeably, estradiol up-regulates cytokeratin levels in BC cells (Coutts et al., 1996; Spencer et al., 1998), and pasireotide may impact cytokeratin levels through activation of signaling networks (Loschke et al., 2015). Under the same experimental conditions, estradiol treatment did not induce significant morphological changes in EPN cells (Figure 5B), and similar findings were observed in cells stimulated with pasireotide, alone or in combination with estradiol (not shown). We then evaluated cell migration by the wound scratch analysis in EPN cells. At 48h treatment, neither estradiol, nor pasireotide, nor combination of both affected cell migration in wound scratch assay. Notably, challenging of cells with PPT and DPN, which specifically activate ERα or β, did not modify the migratory capacity of EPN cells (Figure 5C).

This set of experiments shows that, irrespective of pasireotide presence, estradiol treatment does not promote EMT and migration of EPN cells. In the same experimental conditions, hormonal stimulation of EPN cells activates the apoptotic machinery, and pasireotide enhances this response (see Figure 2, 3).

**FIGURE 6:**
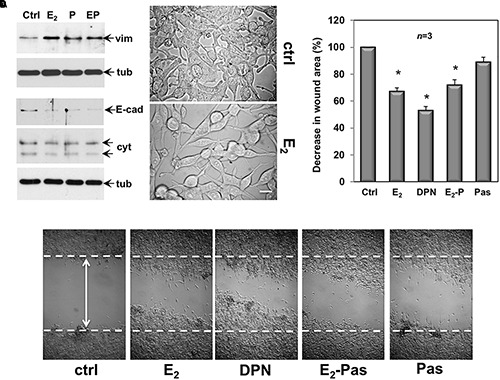
Effect of estradiol and pasireotide on EMT, morphology and motility of CPEC cells. Quiescent CPEC cells were untreated or treated for 48 h with the indicated compounds. Estradiol was used at 20 nM, pasireotide at 0,1 μM and DPN at 3 nM. In panel **(A)**, lysate proteins (2 mg/ml) were prepared, separated by SDS-PAGE and transferred to nitrocellulose membrane. Filters were immune-blotted using the antibodies against the indicated proteins. The blots are representative of two different experiments. In panel **(B)**, the cells were analyzed for morphological changes using contrast-phase microscopy. Bar, 10 μM. In panel **(C)**, the cells were wounded (the arrow indicates the wound length) and then left unstimulated or stimulated with the indicated compounds. Cytosine arabinoside (20 μM) was added to the cell medium to avoid cell proliferation. The wound gap from three different experiments was calculated using Image J software and expressed as % of the decrease in wound area. Means and SEM are shown. *n* represents the number of experiments. ^∗^*p* < 0.05 for each experimental point *versus* the corresponding untreated control. Contrast-phase images in panel **(D)** are representative of 3 different experiments. In panel **(C,D)**, quiescent cells on coverslips were left unstimulated or stimulated for 24 h with estradiol (20 nM), or DPN (3 nM), or pasireotide (0,1 μM) or a combination of estradiol plus pasireotide. After *in vivo* pulse with BrdU (100 μM; Sigma), the DNA synthesis was analyzed by IF as reported in Methods and calculated by the formula: percentage of BrdU-positive cells = (No. of BrdU-positive cells/No. of total cells) X 100. In unstimulated cells, the BrdU incorporation was observed in less than 14% of total cells. Neither estradiol, nor DPN, nor pasireotide, nor combination of estradiol and pasireotide increased the BrdU incorporation, which was detected in 18, 16, 15, and 17% of total cells, respectively.

Quiescent CPEC cells were then used. The cells were left untreated or treated for 48 h with the indicated compounds and lysates were analyzed for expression of vimentin, E-cadherin and cytokeratin. The Western blot in Figure 6A shows that estradiol remarkably up-regulated the vimentin level in CPEC cells. Combination of both, estradiol and pasireotide did not significantly modify such an increase. Up-regulation of vimentin was also observed in pasireotide-stimulated cells. Simultaneously, estradiol decreased E-cadherin and cytokeratin levels. Similar findings were observed by combining both estradiol and pasireotide. A slight decrease in E-cadherin was also observed in cells treated with pasireotide, alone. CPEC cells were then analyzed for morphological changes. The cells appeared elongated and less adherent upon 48h estradiol treatment (Figure 6B). Notably, a simultaneous significant increase in motility was observed upon estradiol challenging of quiescent CPEC cells, as the wound gap was significantly reduced, in the absence of cell proliferation (Figure 6C,D and legend). Addition of pasireotide did not further increase the estradiol-induced effect on cell motility. Although able to induce an increase in vimentin levels and a slight decrease in E-cadherin (Figure 6A), pasireotide did not significantly modify the migratory capacity of CPEC cells, when used alone (Figure 6C,D). Acquisition of a mesenchymal cell state, indeed, is not a prerequisite of a migratory phenotype *in vitro* and *in vivo* (Schaeffer et al., 2014) and pasireotide might be unable, in our experimental conditions, to activate the signaling networks (e.g., Rho family GTPases, paxillin/focal adhesion kinase signaling module) required for cell locomotion (Devreotes and Horwitz, 2015). Notably, stimulation of CPEC cells with the ERβ agonist, DNP strongly reduced the wound gap, indicating that specific activation of ERβ, the only ER isoform expressed in CPEC cells, is responsible for the migratory phenotype here observed (Figure 6C,D).

Collectively, the data in 6 show that by activating ERβ, estradiol promotes EMT and motility in CPEC cells. Addition of pasireotide, or pasireotide alone does not significantly modify these effects. Here again, hormonal stimulation of CPEC cells scantly activates the apoptotic machinery, and pasireotide does not enhance this response (see Figure 2, 3).

In sum, findings in Figure 5, 6 are consistent with the concept that cells undergoing EMT become resistant to the apoptotic effect (Thiery, 2002).

## Discussion

In this study, we have used the prostate epithelial EPN cell line and the PC-derived epithelial CPEC cells. In both cell lines we have assessed the responsiveness to estradiol and the SST analog, pasireotide. Estradiol increases the expression of SSTR1, SSTR2 and SSTR5 at mRNA and protein levels in EPN cells, while hormone treatment reduces the SSTR1 and SSTR2 mRNA and SSTR1, SSTR2 and SSTR5 protein levels in CPEC cells. Our study for the first time reports the regulation of SSTRs by estradiol in prostate cell lines. Estradiol up-regulation of SSTR1, 2 and 5 depends on ERα, since EPN cells express both ERα and β, while CPEC cells only express low amounts of ERβ (Rossi et al., 2011 and Supplementary Figure S2). Experiments with the specific ERα ligand, PPT further support this concept. However, it cannot be excluded the possibility that the balance between the two ER isoforms regulates SSTR levels in EPN cells. In CPEC cells, estradiol down-regulates SSTR1, 2 and 5 likely because of the absence of ERα.

The expression pattern of SSTRs correlates with different biological responses elicited by estradiol. Hormone treatment induces a robust apoptotic response in EPN, but not in CPEC cells, suggesting that up-regulation of SSTR1, 2 and 5 by estradiol is involved in the apoptotic machinery. The finding that estradiol triggers cell death in EPN, but not in CPEC cells, also suggests that the balance between ERα and β expression plays a role in estradiol-induced apoptosis. Loss of this balance, which occurs in CPEC cells, enables cell cycle arrest. The SST analog, pasireotide exerts at very low concentration (0,1 μM) a significant apoptotic effect in both cell lines. The effect is stronger in CPEC cells, as compared with EPN cells, likely because CPEC cells show a more the robust expression of SSTR3, which mainly mediates the SST-induced cytotoxic effect (War et al., 2011). However, other mechanisms (e.g., regulation of PI3-K- or MAPK- or the protein tyrosine phosphatase-dependent pathways or the p27^Kip1^ cell cycle inhibitor) might be involved in CPEC cells. Estradiol reinforces the apoptotic effect by pasireotide only in EPN cells. This finding might be due to the estradiol-induced increases in SSTR2 expression and the consequent apoptotic effect. However, co-expression of both ERα and β might sensitize the EPN cells toward cell death and estradiol activation of both the ER isoforms might be permissive in the apoptotic response.

We observe a down-regulation of Bcl-2 and c-Myc mRNA levels upon estradiol or pasireotide treatment of EPN cells. Combination of estradiol plus pasireotide further increases the Bcl-2 and c-Myc down-regulation. Thus, the apoptotic effect induced by estradiol is mediated by down-regulation of both Bcl-2 and Myc. These findings are consistent with results previously obtained in estradiol-treated EPN cells (Rossi et al., 2011). It has been reported that estradiol up-regulates the Bcl-2 gene in estrogen responsive BC cells (Perillo et al., 2000, 2008). The down regulation of Bcl-2 we observe in EPN cells suggests that estradiol regulation of Bcl-2 depends on cell-context and/or ERα/β ratio. In sum, by increasing the expression levels of SSTRs, estradiol synergizes with pasireotide in prostate cells expressing both ER isoforms. In CPEC cells, which only express low ERβ levels, such regulation is lost and estradiol-treated cells result prevalently arrested in G0/G1. Simultaneously, a significant increase in Bcl-2 level is detected, with a weak elevation in Myc levels, suggesting the involvement of Bcl-2 in estradiol-mediated survival of CPEC cells. It cannot be excluded, however, that a faint elevation in c-Myc levels might amplify pre-existing transcriptional programs (McMahon, 2014).

Conflicting findings on the role of ERα or β in EMT and migration of prostate tissues and PC continue to emerge. Irrespective of pasireotide presence, we did not observe significant morphological changes in estradiol-treated EPN cells. They did not undergo EMT and migration. Noticeably, estradiol induces EMT in prostate benign hyperplasia (Shi et al., 2017). However, the different experimental conditions (2 and 3D conditions), the ratio between ERα and ERβ might explain these discrepancies. In contrast, estradiol challenging of transformed CPEC cells promotes EMT and enables migration. These effects are mediated by ERβ, as CPEC cells only express ERβ and the DPN specific ligand exerts an impressive effect in our migration assay. Overall, our findings are consistent with the concept that apoptosis and motility are mutually exclusive and that migrating cells usually activate survival mechanisms as they move (Hood and Cheresh, 2002). Thus, EPN cells undergo apoptosis, while CPEC cells easily migrate on hormonal stimulation.

The findings we observe call for additional comments. SST analogs, such as octreotide, lanreotide and pasireotide, have been used in PC-derived cells (Lattanzio et al., 2013). Pasireotide, a new synthetic SST analog active on SSTR1-3 and SSTR5 (Weckbecker et al., 2003), induces apoptosis in malignant PC-derived cells and potentiates the antineoplastic effects of prednisone and docetaxel in phase I clinical trial (Lo Nigro et al., 2008; Erten et al., 2009; Thakur et al., 2018). Lanreotide, in combination with dexamethasone and ADT, induces a decrease in PSA level and improves the bone pain in PC patients (Mitsogiannis et al., 2009). Relevant to the findings here reported, combination of SST analogs with ethinyl estradiol restores clinical responses (Di Silverio and Sciarra, 2003) and decreases PSA levels in CRPC patients (Mitsogiannis et al., 2009). Thus CRPC patients benefit from combination of ethinylestradiol and SST analogs in terms of both clinical responses and symptomatic relief. Our findings strongly suggest that combination of SST analogs with estradiol might be efficacious in PC, provided it expresses both the ER isoforms. It cannot be excluded, however, that such combinatorial therapy may act in PC patients by targeting not only PC cells, but also the tumor microenvironment, which confers invasiveness, protection from apoptosis and therapy escape (*reviewed* in Di Donato et al., 2015b). Somatostatin analogs neutralize, indeed, the protective effect elicited by survival signals derived by neuroendocrine prostate cells (Hejna et al., 2002), which often surround PC epithelial cells. On the other, estrogens might exert a direct cytotoxic effect on epithelial PC cells, provided they express both the ER isoforms. Further experiments in 3D models of PC are needed to disclose this concern.

In conclusion, our findings for the first time reports that estradiol controls SSTRs in normal and PC cells. By up-regulating SSTRs, estradiol fosters the anti-proliferative effect of pasireotide in EPN but not CPEC cells. Consistent with these results, estradiol does not promote EMT and motility in EPN cells, while it induces EMT and migration in CPEC cells. In addition to offering valuable hints into the identification of hormonal biomarkers in PC specimens, this study provides new insights in the management of PC, which still remains a challenge for clinicians.

## Author Contributions

VR, LA, and CA contributed to concept and design of study. VR, EDZ, GG, and CDR performed the experiments. VR developed monoclonal antibody panel. EDZ and GG contributed to analysis and representation of data. AS, LA, and CA revised the final draft of the manuscript. GC and AM contributed to writing of the manuscript.

## Conflict of Interest Statement

The authors declare that the research was conducted in the absence of any commercial or financial relationships that could be construed as a potential conflict of interest.

## Footnotes

1
http://imagej.nih.gov/ij/

## Supplementary Material

FIGURE S1 Western blot analysis of protein extracted from normal pancreas with anti-SSTRs monoclonal antibodies (MAbs). Lysate proteins were extracted from normal pancreas and electrophoretically separated as described in Methods. Proteins were then transferred to PVDF membrane and immunoblotted with antibodies against SSTRs receptors (-). Control blots (+) were obtained using anti-SSTRs MAb pre-adsorbed with immune-reactive specific peptide.

FIGURE S2 Western blot analysis of EPN or CPEC lysate proteins with anti-ER (α or β) antibodies. The Western blot analysis of lysate proteins from EPN and CPEC cell lines was done. Proteins from cell lysates (1 mg/ml) were resolved by SDS–PAGE, transferred to nitrocellulose membrane and then probed with anti-ERα or β antibodies, as described in Methods. Lysate proteins from ERα-positive MCF-7 or ERα-negative LNCaP cells were analyzed in parallel, as control. The Western blot analysis with anti-tubulin antibody was also done as loading control.

## Abbreviations

ADT: androgen deprivation therapy
AR: androgen receptor
BC: breast cancer
CRPC: castrate resistant prostate cancer
EMT: epithelial mesenchymal transition
ER: estrogen receptor
PC: prostate cancer
PSA: prostate specific antigen
SST: somatostatin
SSTR: somatostatin receptor
