# Corrigendum: Estrogens modulate somatostatin receptors expression and synergize with the somatostatin analog pasireotide in prostate cells

**DOI:** 10.3389/fphar.2024.1515349

**Published:** 2024-12-02

**Authors:** Valentina Rossi, Erika Di Zazzo, Giovanni Galasso, Caterina De Rosa, Ciro Abbondanza, Antonio A. Sinisi, Lucia Altucci, Antimo Migliaccio, Gabriella Castoria

**Affiliations:** ^1^ Dipartimento di Medicina di Precisione, Università degli Studi della Campania “Luigi Vanvitelli”, Naples, Italy; ^2^ Dipartimento di Scienze Mediche, Chirurgiche, Neurologiche, Metaboliche e dell’Invecchiamento, Università degli Studi della Campania “Luigi Vanvitelli”, Naples, Italy

**Keywords:** prostate cancer, estrogens, somatostatin analogs, somatostatin receptors, apoptosis, EMT, migration

In the published article, there was an error in Figure 5 as published. Figure 5C contained two different pictures representing different fields captured from the PPT-treated cells which had been assembled as two different conditions (PPT and E2+pas, respectively). Figure 5C should only contain an image captured from cells treated with E2+pas. The corrected Figure 5 and its caption appear below.

**FIGURE 5:**
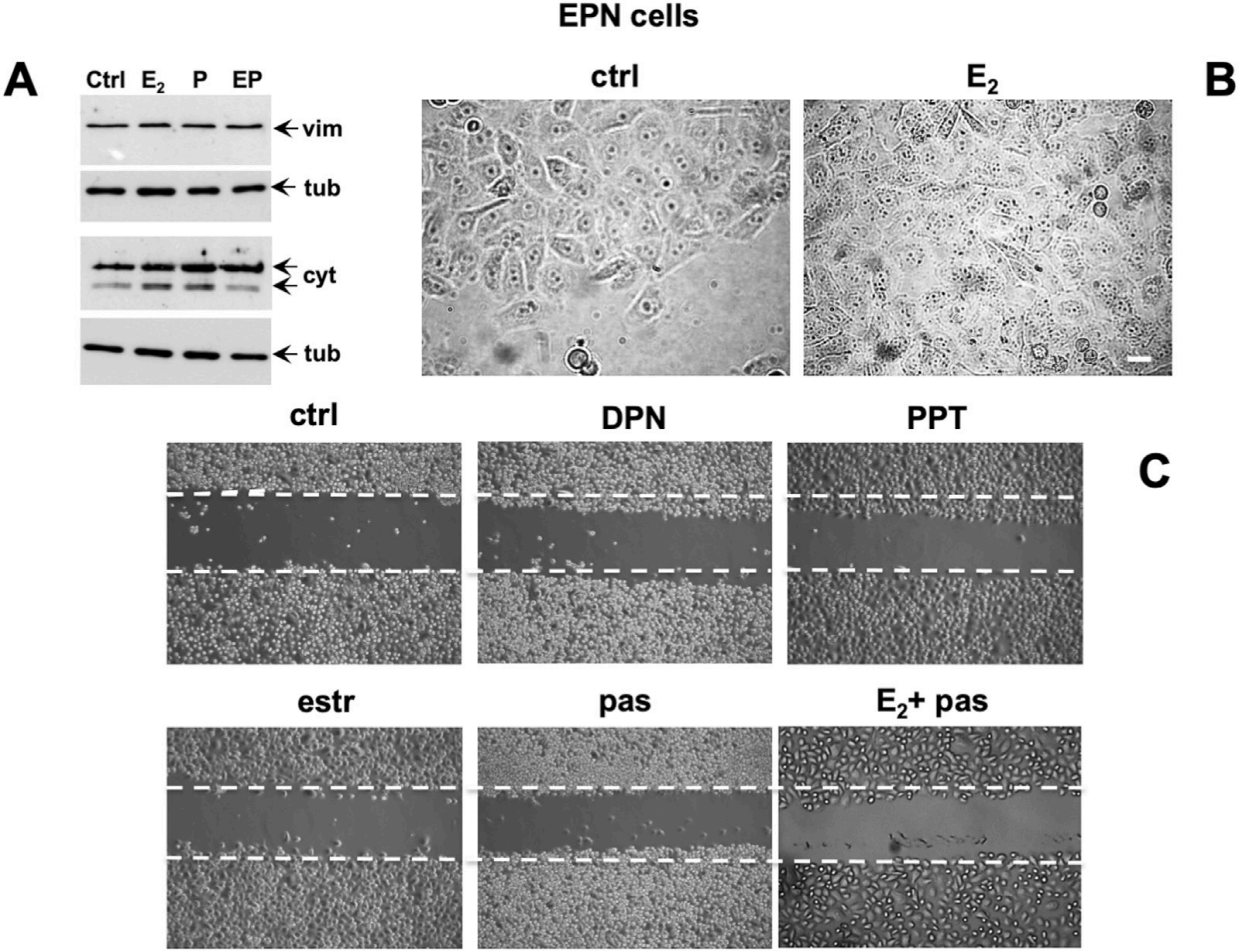
Effect of estradiol and pasireotide on EMT, morphology and motility of EPN cells. Quiescent EPN cells were untreated or treated for 48 h with the indicated compounds. Estradiol was used at 20 nM, pasireotide at 0.1 mM, PPT and DPN both at 3 nM. In panel **(A)**, lysate proteins (2 mg/mL) were prepared, separated by SDS-PAGE and transferred to nitrocellulose membrane. Filters were immune-blotted using the antibodies against the indicated proteins. The blots are representative of two different experiments. In panel **(B)**, the cells were analyzed for morphological changes using contrast-phase microscopy. Bar, 10 mM. In panel **(C)**, the cells were wounded and then left unstimulated or stimulated with the indicated compounds. Cytosine arabinoside (20 mM) was added to the cell medium to avoid cell proliferation. Contrast-phase images in panel **(B, C)** are representative of 3 different experiments.

The authors apologize for this error and state that this does not change the scientific conclusions of the article in any way. The original article has been updated.

