# Supplementary figures and images for: Estrogens Modulate Somatostatin Receptors Expression and Synergize With the Somatostatin Analog Pasireotide in Prostate Cells
Source: Front Pharmacol. 2019 Feb 15;10:28. doi: 10.3389/fphar.2019.00028 (PMC6384260; doi:10.3389/fphar.2019.00028)

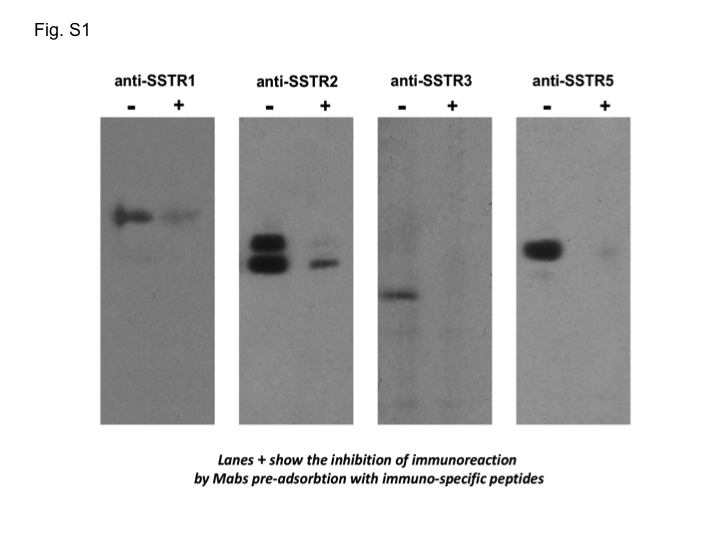

Supplement: FIGURE S1 — Western blot analysis of protein extracted from normal pancreas with anti-SSTRs monoclonal antibodies (MAbs). Lysate proteins were extracted from normal pancreas and electrophoretically separated as described in Methods. Proteins were then transferred to PVDF membrane and immunoblotted with antibodies against SSTRs receptors (-). Control blots (+) were obtained using anti-SSTRs MAb pre-adsorbed with immune-reactive specific peptide. [file Image_1.tiff]

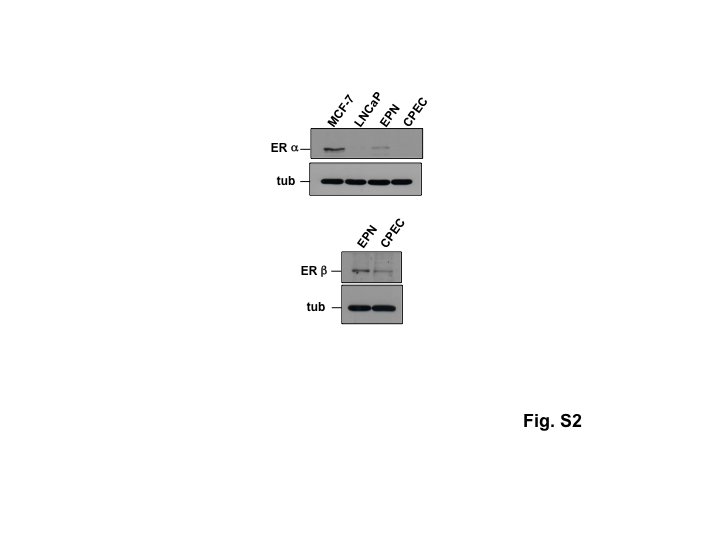

Supplement: FIGURE S2 — Western blot analysis of EPN or CPEC lysate proteins with anti-ER (α or β) antibodies. The Western blot analysis of lysate proteins from EPN and CPEC cell lines was done. Proteins from cell lysates (1 mg/ml) were resolved by SDS–PAGE, transferred to nitrocellulose membrane and then probed with anti-ERα or β antibodies, as described in Methods. Lysate proteins from ERα-positive MCF-7 or ERα-negative LNCaP cells were analyzed in parallel, as control. The Western blot analysis with anti-tubulin antibody was also done as loading control. [file Image_2.tiff]
